# Managing Levothyroxine Malabsorption in Refractory Hypothyroidism: A Case Report

**DOI:** 10.7759/cureus.83123

**Published:** 2025-04-28

**Authors:** Ghada Rashwan, Adil Jumani, Hadiza Ibrahim, Majdi AlNajjar

**Affiliations:** 1 Internal Medicine, Zayed Military Hospital, Abu Dhabi, ARE; 2 Endocrinology, Zayed Military Hospital, Abu Dhabi, ARE

**Keywords:** levothyroxine malabsorption, long levothyroxine absorption test thyroid-stimulating hormone refractory hypothyroidism, overt hypothyroidism, thyroid disorder, thyroid hormone supplements

## Abstract

Levothyroxine malabsorption is a rare but important cause of refractory hypothyroidism that necessitates alternative therapeutic approaches. We present the case of a 55-year-old woman with long-standing hypothyroidism who continued to experience persistent symptoms despite being on high-dose oral levothyroxine (1500 mcg daily). Laboratory investigations revealed an elevated thyroid-stimulating hormone (TSH) level of 14.160 mIU/L, along with positive anti-gliadin and anti-parietal cell antibodies. A supervised thyroxine (T4) absorption test confirmed true levothyroxine malabsorption. The patient was subsequently transitioned to a liquid levothyroxine formulation at a reduced dose of 600 mcg, which led to significant clinical improvement and normalization of thyroid function. Levothyroxine malabsorption can be attributed to underlying gastrointestinal conditions such as celiac disease and atrophic gastritis. The T4 malabsorption test is a valuable tool in distinguishing true malabsorption from non-adherence or pseudo-malabsorption. In cases of confirmed malabsorption, liquid or parenteral formulations may help improve absorption and lead to better clinical outcomes. This case highlights the importance of identifying thyroid hormone malabsorption in patients with refractory hypothyroidism to ensure optimal management and therapeutic success.

## Introduction

Hypothyroidism is a condition characterized by insufficient production of thyroid hormone due to dysfunction of the thyroid gland. This deficiency can arise from various causes, with autoimmune disease being the most prevalent cause in iodine-sufficient areas [1]. Prevalence of hypothyroidism ranges between 0.1% and 2% [2,3], varying by age and gender, with a higher incidence observed in females [3]. Often, hypothyroidism is subclinical [4], being picked up on routine testing.

Hypothyroidism is typically treated with an oral synthetic thyroxine (levothyroxine) with dosages generally ranging from 1.6 to 1.8 µg/kg [1], tailored based on several factors, including age, body weight, pregnancy status, and presence of heart disease, among others. However, some patients may not respond to oral thyroxine despite being on adequate doses due to factors affecting thyroxine absorption. Key factors include interactions with food, medications (such as proton pump inhibitors, antacids, calcium supplements), and the presence of underlying malabsorptive conditions such as celiac disease, lactose intolerance, inflammatory bowel disease (IBD), and *Helicobacter pylori* infection [5]. Therefore, it is crucial to review patients’ medication schedules and concurrent use of other drugs when assessing inadequate response to therapy. Pseudo-malabsorption must also be considered, often arising from medication nonadherence or unrecognized drug interactions. Refractory hypothyroidism is typically defined as requiring more than 1.9 mcg/kg/day of levothyroxine without achieving target thyroid-stimulating hormone (TSH) levels [6].

Gastrointestinal disorders impair levothyroxine absorption through several mechanisms. For example, villous atrophy in celiac disease reduces the absorptive surface area available for hormone uptake, while chronic atrophic gastritis alters the gastric pH required for optimal levothyroxine dissolution and absorption [5]. As a result, even with appropriate dosing, absorption may remain suboptimal in affected patients.

When refractory hypothyroidism persists despite addressing common interfering factors, a supervised thyroxine (T4) absorption test becomes a valuable diagnostic tool to differentiate true malabsorption from pseudo-malabsorption related to nonadherence or drug interactions [7]. Early identification allows for tailored management, including the use of alternative formulations. Liquid and parenteral levothyroxine preparations offer improved absorption profiles, bypassing the need for gastric dissolution, and are particularly effective in patients with gastrointestinal dysfunction [8,9].

In some circumstances, patients may continue to exhibit persistent hypothyroid symptoms and abnormal thyroid function tests despite addressing these factors and receiving high doses of oral levothyroxine. This article presents such a case, highlighting the diagnostic and therapeutic challenges encountered.

## Case presentation

We report a case of a 55-year-old female with a history of long-standing hypothyroidism who presented to the endocrinology clinic for management of persistent hypothyroid symptoms. The patient had been diagnosed with hypothyroidism 19 years prior. She had experienced persistent symptoms of fatigue, weight gain, and dry skin throughout the 19 years since her initial diagnosis.

She had been on large levothyroxine doses (475 mcg daily), approximately three times the standard dosage based on her weight (1.6 mcg/kg, equivalent to 168 mcg). Despite this, she continued to experience symptoms.

Laboratory tests done at the time of the clinic visit revealed significantly elevated TSH levels (14.610 mIU/L; reference range: 0.270-4.20 mIU/L) and low free T4 levels (8.64 pmol/L). On physical examination, the patient was overweight with a body mass index (BMI) of approximately 38 kg/m^2^. No other significant abnormalities were noted. The patient reported strict adherence to her prescribed levothyroxine regimen.

The patient was advised on proper medication administration, including avoiding interactions with food and other drugs that could affect absorption. Her levothyroxine dose was gradually increased every four to six weeks, with close monitoring of TFTs, eventually reaching 600 mcg daily. Despite these adjustments, the patient’s TFTs remained abnormal, and she continued to experience symptoms.

A gastroenterology consultation was sought to investigate possible underlying malabsorptive conditions. Workup for celiac disease, pernicious anemia, *H. pylori* infection, and small bowel intestinal overgrowth was performed. Laboratory results showed positive anti-gliadin antibodies (20.6 U/mL) and positive anti-parietal cell antibodies (titer >1:10). The patient was started on intramuscular cyanocobalamin 1000 mcg weekly. Endoscopy revealed no macroscopic abnormalities; however, biopsies taken from the stomach and duodenum showed mild chronic superficial gastritis, with a negative Campylobacter-like organism (CLO) test, normal duodenal villi, and no evidence of metaplasia, dysplasia, or malignancy.

Despite close follow-up and continued levothyroxine dose escalation, reaching up to 1500 mcg daily, the patient’s TFTs and symptoms remained poorly controlled. Therefore, a supervised T4 absorption test was conducted to evaluate for true levothyroxine malabsorption.

**Figure 1:**
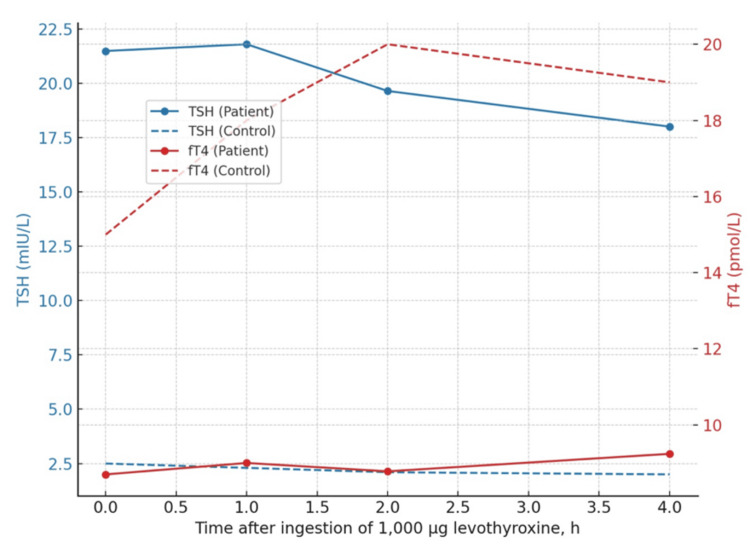
Trends in the patient’s thyroid-stimulating hormone (TSH; mIU/L) and free thyroxine (free T4; pmol/L) levels following supervised ingestion of 1000 micrograms (mcg) of levothyroxine. Minimal rise in free T4 and persistently elevated TSH confirmed impaired levothyroxine absorption. Control values represent expected levels from healthy individuals. In healthy subjects, free T4 typically rises significantly after levothyroxine ingestion. TSH and free T4 units are expressed on the Y-axes in mIU/L and pmol/L, respectively.

The patient fasted overnight prior to the absorption test. She was administered 1000 mcg of T4 under direct supervision, and serum TSH was measured at intervals zero, one, and two hours. Free T4 was measured at 30, 45, 60, 120, 180, and 240 minutes (Figure 1).

Trends observed during the supervised T4 absorption test are shown in Figure 1. In contrast to healthy individuals, the patient’s free T4 levels showed only minimal increase over time, and TSH remained persistently elevated, consistent with true levothyroxine malabsorption. During the absorption test, the patient’s free T4 concentrations rose minimally from a baseline of 8.70 pmol/L to a peak of 9.24 pmol/L at 240 minutes, confirming impaired levothyroxine absorption.

The test confirmed true levothyroxine malabsorption, and the patient was subsequently switched to oral liquid levothyroxine. At a dose of 600 mcg daily, her TFTs normalized within six weeks, accompanied by significant symptom resolution.

## Discussion

This case describes the presence of refractory hypothyroidism in a female with longstanding hypothyroidism secondary to underlying thyroxine malabsorption confirmed by a T4 malabsorption test. Refractory hypothyroidism is a challenging condition characterized by persistently elevated TSH levels despite adequate daily doses of oral levothyroxine, typically defined as requiring more than 1.9 mcg/kg/day [6]. Although the precise prevalence remains undetermined, it represents a significant clinical challenge for both the healthcare provider and the patient.

The presence of persistently elevated TSH levels despite high doses of levothyroxine often necessitates a thorough evaluation of potential underlying causes. A levothyroxine malabsorption test is important to distinguish true malabsorption from "pseudo-malabsorption" [7].

In patients with confirmed levothyroxine malabsorption, switching to alternative formulations of levothyroxine can improve absorption and clinical outcomes. The use of liquid levothyroxine has been shown to be particularly effective. A meta-analysis by Laurent et al. compared the efficacy of liquid versus tablet levothyroxine and found that patients with documented malabsorption who were switched to liquid levothyroxine exhibited notable improvements in their TFTs [8]. Liquid formulations of levothyroxine provide more consistent and predictable absorption, especially in patients with compromised gastrointestinal absorption.

In addition to liquid formulations, other studies have explored the use of parenteral levothyroxine. Groener et al. investigated the efficacy of subcutaneous levothyroxine in patients with severe levothyroxine malabsorption who required excessively high oral doses. Their study demonstrated significant improvements in both laboratory results and clinical symptoms within two weeks of initiating subcutaneous therapy [9]. However, while effective, it is less commonly used, likely due to associated complexities and potential patient discomfort.

The decision to switch to a different formulation depends on various factors, including the severity of malabsorption, patient preference, and drug form availability. Liquid levothyroxine, due to its ease of use and lower risk of discomfort, may be preferred as a first-line alternative.

This case highlights the importance of recognizing and addressing levothyroxine malabsorption in patients with refractory hypothyroidism. The successful management of such cases often requires a shift from standard oral levothyroxine to alternative formulations or different routes of administration. Future research should focus on determining the prevalence of levothyroxine malabsorption across different patient populations and refining treatment protocols. Additionally, studies comparing the long-term efficacy and patient satisfaction of liquid versus parenteral levothyroxine could provide valuable insights for optimizing treatment strategies.

## Conclusions

This case highlights the importance of considering levothyroxine malabsorption in patients with refractory hypothyroidism. In our patient, minimal free T4 rise during a supervised T4 absorption test confirmed true malabsorption. Transitioning to a liquid levothyroxine formulation at a reduced dose of 600 mcg led to normalization of TFTs within six weeks and significant clinical symptom improvement. Early identification of malabsorption and timely initiation of alternative formulations, such as liquid thyroxine, can optimize patient outcomes and reduce unnecessary treatment escalation.
